# Oxygen regulates ILC3 antigen presentation potential and pregnancy-related hormone actions

**DOI:** 10.1186/s12958-022-00979-2

**Published:** 2022-07-29

**Authors:** Rebekka Einenkel, Jens Ehrhardt, Marek Zygmunt, Damián Oscar Muzzio

**Affiliations:** 1 Department of Obstetrics and Gynecology, University Medicine Greifswald, Greifswald, Germany; 2 Present address: Gynecologic Endocrinology and Reproductive Medicine, University Hospital Bonn, Bonn, Germany

**Keywords:** Innate lymphoid cells, Oxygen, hCG, TGF-β1, Pregnancy, Tolerance, Placenta

## Abstract

Early pregnancy is marked by placentation and embryogenesis, which take place under physiological low oxygen concentrations. This oxygen condition is crucial for many aspects of placentation, trophoblast function, vascularization and immune function. Recently, a new family of innate lymphoid cells has been found to be expressed at the fetomaternal interface. Among these, type 3 innate lymphoid cells (ILC3) are important antigen presenting cells in the context of MHC-II. The expression of MHC-II on ILC3s during pregnancy is reduced. We tested the hypothesis that low oxygen concentrations reduce the potential of ILC3s to present antigens promoting fetal tolerance.

Using an in vitro approach, NCR^+^ ILC3s generated from cord blood stem cell precursors were incubated under different O_2_ concentrations in the presence or absence of the pregnancy-related hormones hCG and TGF-β1. The expression of MHC-II, accessory molecules and an activation marker were assessed by flow cytometry. We observed that 1% O_2_ reduced the expression of the MHC-II molecule HLA-DR as compared to 21% O_2_ and modulated the relative effects of hCG and TGF-β1.

Our data indicate that low oxygen concentrations reduce the antigen presentation potential of NCR^+^ ILC3s and suggest that it may promote fetal tolerance during the first trimester of pregnancy.

**Supplementary Information:**

The online version contains supplementary material available at 10.1186/s12958-022-00979-2.

## Introduction

After implantation, several changes take place in the decidualized endometrium to assure the anchoring of the fetus and the later establishment of a fetomaternal circulation. Trophoblast cells invade into the maternal decidua, reach spiral arteries and block them temporarily. During this time, the zone between decidua and placenta remains in low oxygen conditions, which is estimated to be around 10–30 mmHg (corresponding to 1–4% O_2_) [1, 2]. Once the plugs are released, the intervillous spaces are filled with maternal blood. Thereafter, the oxygen partial pressure reaches 50–60 mmHg (7–8% O_2_) [2]. The reduced oxygen availability promotes placentation by inducing cell growth and vascular remodeling [3]. Moreover, higher O_2_ concentrations are suspected to be detrimental to early pregnancy and have been recorded in missed abortions [2, 4].

The adaptations are supported by local factors as TGF-β1 and hCG [5–8]. The changes in the O_2_ concentrations also influence the function of several cell types, including leukocytes that are present at the fetomaternal interface [9]. As it occurs with trophoblasts and endothelial cells, also decidual leukocytes (including macrophages and innate lymphoid cells (ILCs) as NK cells) take part in vascular remodeling are influenced by low oxygen concentrations [10].

The role of lymphocytes in vascular remodeling is not completely understood. Recently, new lymphocyte populations with angiogenic potential have been described. In particular, the helper subsets of the ILCs, which are known to be involved in tissue remodeling [11]. ILCs secrete diverse cytokines (e.g. IL-8, IL-17, IL-22, IFNγ, GM-CSF) and influence the function of other cells, including T cells. As excessive cytokine secretion may disturb placentation, dysregulated secretory capacity of immune cells and their interactions at the fetomaternal interface are of great importance.

The interaction between ILCs and T cells is greatly dependent on cell-to-cell contacts. ILCs express PD-1, OX40L, BAFF, DLL1, CD80 and CD86, which play an important role in the activation and modulation of adaptive immune cells [12–14]. Therefore, ILCs represent an important link between adaptive and innate immune responses. Particularly, the antigen presentation capacity of ILCs has been found to be fundamental in immune homeostasis, as for instance in the gut [12, 15, 16]. The expression of antigen-presentation markers and accessory molecules on ILCs at the fetomaternal interface has not yet been fully characterized [17]. In a recent work, we have observed that the antigen presentation potential of ILCs is reduced during pregnancy [18]. Furthermore, the antigen presentation potential of ILCs was restricted at the fetomaternal interface, and there were differences between decidua basalis and decidua parietalis concerning the expression of HLA-DR. This phenomenon may represent an adaptive mechanism involved in the tolerance against fetal antigens or an excessive inflammatory response against viral or bacterial derived molecules. In this sense, ILCs at the fetomaternal interface are characterized by a cytokine secreting phenotype with restricted antigen presentation capacity.

The mechanisms for tolerance against fetal antigens include the absence of classical MHC-I and MHC-II expression by trophoblast cells [19–21]. CD4^+^ T cells, however, recognize fetal antigens indirectly, in a process mediated by antigen presenting cells (APC). APC at the fetomaternal interface include macrophages, dendritic cells, B cells and ILCs [22].

Physiological oxygen restriction is known to affect immune cells and lead, as in the case of the placenta, to tolerance-mediating effects [23]. Similar to first trimester placenta, a low oxygen concentration is characteristic of the tumor environment. The low oxygen availability of the tumor environment dampers the anti-tumoral responses of several immune cells [10]. The effects include a reduction of the expression of MHC-II molecules [24, 25]. Here, we postulate that the low oxygen conditions that prevail at the beginning of pregnancy promote a tolerogenic phenotype in ILCs.

## Methods

### Human samples

Umbilical cord blood of term cesarean sections was obtained after patient’s informed consent. Mononuclear cells were isolated as previously described [18]. Briefly, blood samples were centrifuged, serum discarded and cells were isolated by density centrifugation with Lymphoprep™ (STEMCELL Technologies Inc., Vancouver, Canada), following manufacturer’s instructions.

### Cell culture

Umbilical cord blood cells were used as source of stem cells, which were isolated applying CD34 MicroBead Kit UltraPure, human (Miltenyi, Bergisch Gladbach, Germany). According to manufacturer’s handbook, CD34^+^ stem cells were isolated by magnetic separation. Therefore, cells were incubated with Fc Block and microbead-coupled anti-CD34 antibody for 15 min at 4 °C. After washing, CD34^+^ cells were isolated using magnetic MS column. Bound cells were flushed after removing from the magnet.

Stem cells were then cultured in ILC3 differentiation medium (10% FBS, 1% Penicillin-Streptomycin (PAN-Biotech GmbH, Aidenbach, Germany), 20 ng/mL SCF, 20 ng/mL IL-7, 20 ng/mL IL-15 and 10 ng/mL Flt-3 ligand (Miltenyi, Bergisch Gladbach, Germany) in RPMI 1640 (PAN-Biotech GmbH, Aidenbach, Germany)) as previously reported [18, 26, 27]. After 30 days of culture, an ILC3-enriched culture was obtained. These cells were used for further experiments.

Treatment was performed in the presence of TGF-β1 (2 ng/mL (R&D, Minneapolis, MN, USA)) or hCG (100 IU/mL (Ovitrelle®, Merck, Darmstadt, Germany)). Both concentrations were chosen for being in physiological range in serum and to better compare data with own and others previous studies [18, 28–34]. Treatment under different oxygen concentrations (1 or 8% O_2,_ mimicking first and second trimester oxygen availability at the fetomaternal interface, respectively) was performed in a multigas incubator (Sanyo, Moriguchi, Osaka prefecture, Japan) supplied with 5% CO_2_ and N_2_ as complementary gas. In parallel, cells were cultured in an identical multigas incubator under standard culture conditions (21% O_2_, 5% CO_2_ and N_2_ as complementary gas) as controls. CO_2_ and O_2_ concentrations were measured constantly. Cells were activated with human recombinant IL-1β and IL-23 (20 ng/mL (BioLegend, San Diego, CA, USA)) for the last 18 h of the stimulation. An increased signal of intranuclear HIF-1α under 1% O_2_ was observed by flow cytometry after nuclei isolation and further staining with anti-HIF-1α antibody (BD Biosciences, Franklin Lakes, NJ, USA; clone 54/HIF-1α; data not shown).

### Flow cytometry

Cell suspensions were preincubated with Fixable Viability Dye (Thermo Fisher Scientific Inc., Waltham, MA, USA) for 30 min at 4 °C to exclude non-viable cells. For subsequent washing steps FACS buffer (1% BSA (Sigma-Aldrich, St. Louis, MO, USA), 0.1% NaN_3_ (Carl Roth, Karlsruhe, Germany) in DPBS (PAN-Biotech GmbH, Aidenbach, Germany)) was used. Extracellular staining was performed for 30 min at 4 °C. For intracellular staining cells were permeabilized with Foxp3 staining buffer set (Thermo Fisher Scientific Inc., Waltham, MA, USA) according to manufacturer’s instructions. Intracellular staining was applied for 30 min at 4 °C as well.

Antibody clones used for flow cytometry staining from BD Biosciences (Franklin Lakes, NJ, USA): Lin3 (CD3, MφP9; CD14, SK7; CD19, SJ25C1; CD20, L27), CD94 (HP-3D9), NKp44 (p44–8), CD80 (L307.4), CD40 (5C3), CD69 (FN50) and from Miltenyi (Bergisch Gladbach, Germany): NKp44 (Clone 2.29), HLA-DR (AC122).

Measurement was performed with FACSCanto (BD Biosciences, Franklin Lakes, NJ, USA). Immediately before measurement, counting beads (BD Biosciences, Franklin Lakes, NJ, USA) for quantification were added. FMO (fluorescence minus one) controls were included. Data was analyzed with FlowJo10.4 software (FlowJo, LLC, Ashland, TN, USA).

NCR^+^ ILC3s were gated as live CD3^−^CD14^−^CD19^−^CD20^−^CD94^−^NKp44^+^ cells. Cell count was determined by addition of counting beads, which were gated separately. The sum of NCR^+^ ILC3s was calculated by the measured cell number times the added beads divided by the measured beads. The identity of the cells was confirmed as in our previous study [18]. As shown, after sorting and subsequent stimulation, these cells express CD69, HLA-DR, IL-8 and IL-22. The presence of RORγt, HLA-DR, CD80, CD40, CD69, IL-8 and IL-22 mRNA was also detected by qPCR and flow cytometry [18]. Additionally, IL-8, IL-17 and IL-22 were detected in the supernatants of sorted cells (Bio-Plex Pro™ Human Th17 Cytokine Assay (Bio-Rad, Hercules, California, USA). As the data presented in this manuscript was obtained by flow cytometry from enriched ILC3s, qPCR and Multiplex data is not shown. The gating strategy for the sorting, the purity assessment and the characterization of sorted NCR^+^ ILC3s by flow cytometry is shown in the Supplementary figure.

### Statistical analysis

Data are presented as relative changes to corresponding untreated controls. Values were normalized to the mean of the untreated controls to set them 1 and keep the variances of both, treated and untreated cells. Paired Student’s *t*-test was used to analyze data. Graphs show mean ± SEM. For statistical analysis Microsoft Excel 2010 (Microsoft, Redmond, WA, USA) and Prism 5.01 (GraphPad Software, San Diego, CA, USA) were used. *P*-value ≤0.01 was considered statistically significant.

## Results

### Oxygen restriction reduces ILC3 antigen presentation potential

Recently, we showed that antigen presentation capacity by ILC3s is reduced in uterine and decidual compared to peripheral samples [18]. Since conditions present at the fetomaternal interface as soluble factors and low oxygen concentration can shape lymphocytes into a decidual like phenotype [35], we started by evaluating the role of low oxygen concentrations on ILC3 antigen presentation potential.

**Fig. 1:**
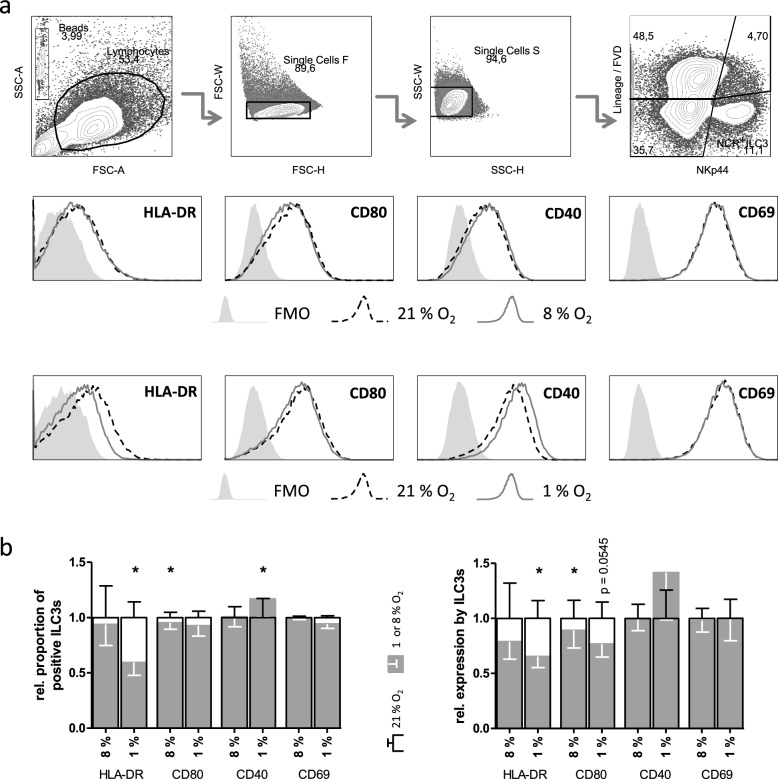
Oxygen restriction reduces ILC3 antigen presentation potential. In vitro-generated ILC3s were incubated under indicated oxic conditions for 72 h. Cells were activated with 20 ng/mL IL-1β and IL-23 for 18 h prior flow cytometry staining. **A** Representative gating strategies for identification of NCR^+^ ILC3s are shown. The overlapping histograms depict the expression of extracellular markers on NCR^+^ ILC3s under 21% O_2_ (stripped line) or reduced oxygen concentration (filled line). FMO controls are displayed as a filled curve. **B** Graphs show percentage of marker-expressing ILC3s (left) and the mean fluorescence intensity (MFI; right) of HLA-DR, CD80, CD40 and CD69. Data of 8 or 1% O_2_ (grey) was normalized to 21% O_2_ (outlined bar). Data is shown as mean ± SEM and was analyzed by paired Student’s *t*-test. *p < 0.05. Experiment was performed for each condition 5 times in duplicates

To test the effect of oxygen-restricted conditions on ILC3s, in vitro-generated NCR^+^ ILC3s were incubated in the presence of atmospheric oxygen concentration (21% O_2_), and parallelly either under 8% O_2_ or 1% O_2_ (Fig. 1).

Compared to 21% O_2_, the incubation in 1% O_2_ significantly reduced the percentage of HLA-DR^+^ ILC3s (by 41%) and their antigen presentation capacity (MFI; by 35%). Costimulatory molecules CD40 and CD80 were contrary affected by reduced O_2_ concentration. Under 8% O_2_, the proportion of CD80^+^ ILC3s and the CD80 MFI were significantly decreased (by 11 and 9%, respectively) compared to 21% O_2_. In contrast, the percentage of CD40^+^ ILC3s was significantly increased (by 17%) under 1% O_2_ compared to 21% O_2_.

The activation marker CD69 was not significantly affected by oxygen restriction.

### Oxygen modulates hCG-mediated effects on ILC3s

**Fig. 2:**
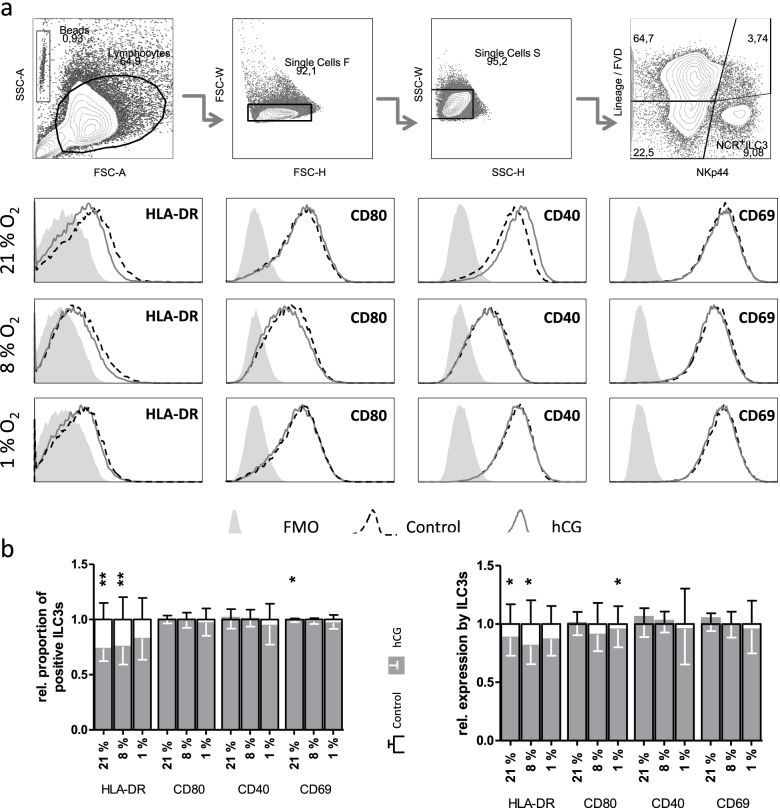
hCG addition complements antigen presentation potential reduction in ILC3s. Additionally to oxic conditions, in vitro-generated ILC3s were stimulated with 100 IU/mL hCG for 72 h. 18 h before flow cytometry staining cells were activated with 20 ng/mL IL-1β and IL-23. **A** Representative gating strategies for identification of NCR^+^ ILC3s are shown. The overlapping histograms depict the expression of extracellular markers on NCR^+^ ILC3s under 21% O_2_ (stripped line) or reduced oxygen concentration (filled line). FMO controls are displayed as a filled curve. **B** The proportion of marker-expressing ILC3s (left) and the MFI of HLA-DR, CD80, CD40 and CD69 were analyzed (right). Effects of hCG (grey bars) were normalized to untreated cells (outlined bars) under the same oxic conditions. Bars show mean ± SEM. Data was analyzed by paired Student’s *t*-test. *p < 0.05, **p < 0.01. The experiment was repeated 5 (8 and 1% O_2_) or 10 times (21% O_2_) in duplicates

Pregnancy-related hormones hCG and TGF-β1 reduce ILC3 antigen presentation potential [18]. Since lower oxygen concentration reduced antigen presentation capacity, a synergistic effect of hCG was assessed (Fig. 2).

HCG significantly reduced the percentage of HLA-DR^+^ ILC3s and HLA-DR expression (MFI) under 21% O_2_ (by 26 and 12%, respectively) and 8% O_2_ (by 24 and 19%, respectively). The effect under 1% O_2_ was not statistically significant.

Oxygen also modulated hCG-mediated effects on CD80 expression, leading to a decrease in CD80 expression after treatment with hCG under 1% O_2_ (by 5%). However, CD40 expression was not additionally affected by hCG treatment. The expression of CD69 was significantly reduced by hCG under 21% O_2_, but only by 1%.

### ILC3s under 1% O_2_ are more sensitive to TGF-β1-mediated reduction of CD40 and CD69

**Fig. 3:**
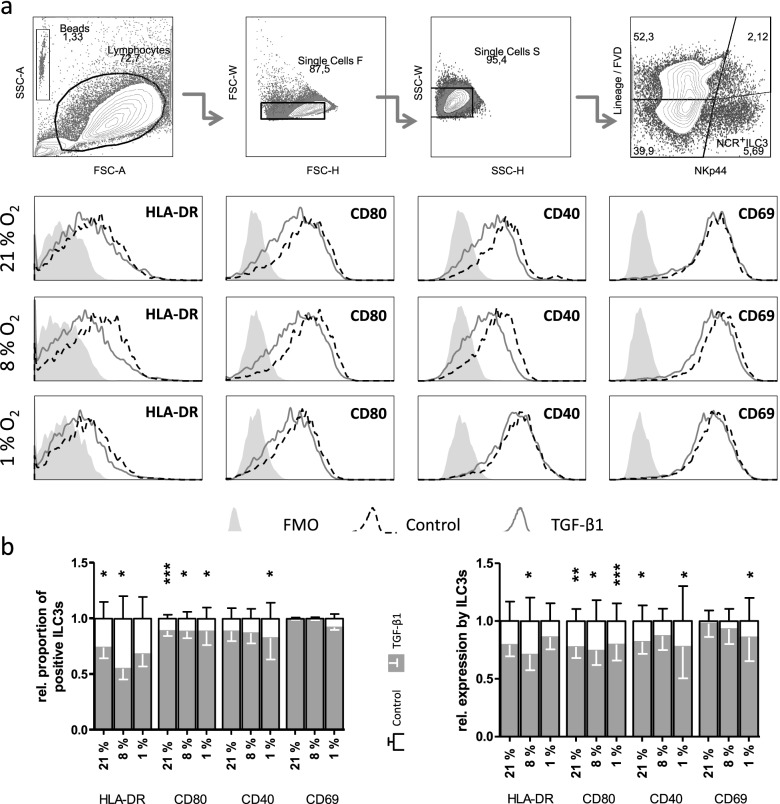
Reduced oxygen availability enhances TGF-β1-mediated reduction in ILC3 accessory molecules. Defined oxic conditions were combined with the addition of 2 ng/mL TGF-β1 to treat in vitro-generated ILC3s for 72 h. Cells were activated by 20 ng/mL IL-1β and IL-23 18 h before flow cytometry staining. **A** Representative gating strategies for identification of NCR^+^ ILC3s are shown. The overlapping histograms depict the expression of extracellular markers on NCR^+^ ILC3s under 21% O_2_ (stripped line) or reduced oxygen concentration (filled line). FMO controls are displayed as a filled curve. **B** The proportion of ILC3s expressing the indicated marker (left) and its MFI (right) were analyzed. Effect of TGF-β1 (grey bars) is shown relative to controls with the same oxic condition (outlined bars). Bars show mean ± SEM. Data was analyzed by paired Student’s *t*-test. *p < 0.05, **p < 0.01, ***p < 0.001. The experiment was repeated 5 (8 and 1% O_2_) or 10 times (21% O_2_) in duplicates

Next, additive effects of O_2_ restriction and TGF-β1 were investigated, by comparing TGF-β1-treated and untreated cells (Fig. 3).

TGF-β1 reduced the proportion of HLA-DR-expressing ILC3s under 21% (by 27%) and under 8% O_2_ (by 46%), but not significantly under 1% O_2_. HLA-DR expression (MFI) of ILC3s was significantly reduced through TGF-β1 only under 8% O_2_ (by 29%).

CD80 expression and proportion of CD80^+^ ILC3s was significantly decreased by TGF-β1 under all oxygen conditions (percentage: by 12% each; MFI: by 22, 26, 20%, respectively).

Although oxygen restriction to 1% O_2_ increased the proportion of CD40^+^ ILC3s, stimulation with TGF-β1 significantly reduced CD40 expression and the proportion of CD40-expressing ILC3s under 21% (by 18%; 12%) and 1% O_2_ (by 22, 18%).

CD69 expression became more sensitive to TGF-β1-mediated regulation with decreasing O_2_ concentrations. TGF-β1 did not significantly change CD69 expression under 21 or 8% O_2_, but significantly reduced CD69 expression by ILC3s under 1% O_2_ (by 14%).

## Discussion

The role of oxygen on ILC biology has not been studied extensively [36]. In several tissues, oxygen modulates diverse biological processes, especially angiogenic events [10]. During placentation, the trophoblastic plug in the spiral arteries leads to a temporary state of low oxygen availability in the placenta and the contact zone of the decidua [1]. At this time, important modifications of the vasculature and the extracellular matrix take place [37] and the interplay between trophoblast cells and several immune cell subsets is crucial [38].

The fact that fetal cells (expressing paternal antigens) are tolerated by maternal immune system represents a significant challenge to our understanding of immune response mechanisms. Nevertheless, it is well-accepted that immune cells adapt during pregnancy to avoid fetal rejection [39, 40]. The tolerance to fetal antigens comprises both, active and passive mechanisms. Active mechanisms of fetal tolerance include cellular and humoral components which are triggered after paternal antigen recognition. In 1–5% of pregnancies, nonetheless, early spontaneous recurrent pregnancy loss can be observed. In the absence of miscarriage inducing factors, idiopathic recurrent pregnancy losses are believed to be caused mainly by immune factors; particularly, the impaired recognition of paternal antigens and the subsequent loss of control over the inflammatory response [41].

Trophoblasts are invasive fetal cells that establish close contact with maternal immune cells [38]. In order to avoid immune rejection, trophoblasts do not express most polymorphic classical MHC-I and MHC-II molecules. MHC-II molecules, however, can be found in intracellular deposits of trophoblast cells [42]. During placentation, trophoblasts undergo cycles of growth and apoptosis [43]. Antigen presenting cells clean up the debris and can in this context present fetal antigens to CD4^+^ T cells [44]. In order to induce tolerance, instead of fetal rejection, the antigen presentation should occur in a manner that induces T cell tolerance instead of a pro-inflammatory phenotype.

ILC3s are central components of immune homeostasis in mucosal barriers, where they collaborate with the tolerance against commensal bacteria [16, 45, 46]. ILC3s can intake and process antigens, to later present them in the context of MHC-II to CD4^+^ T cells [47, 48]. This interaction plays a fundamental role in the induction of intestinal commensal bacteria tolerance [49]. Although altered ILCs numbers have been described in disturbed pregnancies, the role of ILCs in fetal tolerance has not been studied extensively yet [50–55]. Our group has previously observed that ILCs reduce their antigen presentation potential during pregnancy, and that uterine ILCs retain a low presentation potential [18]. Additionally, local soluble factors can modulate antigen presentation potential. Here we observed that low oxygen concentrations, as measured in the contact zone between decidua and placenta, induce a reduction of the MHC-II expression. This can be interpreted as induction of tolerance, which may facilitate the invasion of fetal cells into the maternal decidua.

Furthermore, we observed that the effects of factors that regulate ILC3 function, such as hCG or TGF-β1, are also dependent on the oxygen availability. The drastic reduction of MHC-II expression in the culture at 1% O_2_ seemed to mask the relative effects of hCG and TGF-β1. These, on the other hand, may play an important role later on in pregnancy, when the blood flow into the fetal circulation has been established and the oxygen concentration increases.

The modulatory role of oxygen could be appreciated in the expression of co-stimulatory molecules as well. The culture under 1% O_2_ had an additive effect on the hCG modulation of the expression of CD80 and improved the regulatory effect of TGF-β1 on CD40 and CD69.

Our results support the idea that local factors at the fetomaternal interface can shape ILC3 function into a tolerogenic phenotype in terms of antigen presentation. These results encourage further research to elucidate how ILC3s shape their decidual phenotype and determine a cytokine producing, low antigen presentation potential. The influence of further soluble factors, cell-to-cell contact and a possible distinct local in situ development should be considered in future research.

The maintenance of fetal tolerance is fundamental for pregnancy success. The loss of fetal tolerance can induce pathological conditions as in villitis of unknown etiology and secondary recurrent miscarriage [56]. It has been observed that alterations of the oxygenation at the beginning of pregnancy impact negatively on pregnancy outcome. Premature or excessive entry of maternal blood into the intervillous spaces is observed in miscarriage [4, 57]. The deleterious effects on fetal cell function of the resulting over oxygenation are partially mediated by free oxygen radicals, which impair early pregnancy events [58, 59]. It is likely that the downregulation of the antigen presentation potential of ILC3s by low oxygen contributes to early tolerance against fetal-derived antigens. Alternatively, a regulated antigen presentation could be fundamental to avoid excessive inflammatory responses towards microbiota derived antigens. This mechanism might represent a physiological adaptation in the context of the presence of non-infective bacteria as suggested by microbiome research in the upper reproductive tract [60].

During the first trimester of pregnancy, which is characterized by physiological low oxygen concentrations in the placenta, trophoblasts possess an invasive phenotype. There is, as a consequence, an increased cell replication which is balanced by a high apoptotic rate [61]. It has been proposed that antigen presenting cells clean up apoptotic trophoblasts [62]. For this reason, it is tempting to speculate that a reduced fetal antigen presentation would positively influence the balanced immune responses at the fetomaternal interface, by preventing excessive immune reactions. On the contrary, as observed in hyperoxic placentas, pregnancy problems may arise.

## Supplementary Information

**Additional file 1: **Supplementary figure. The identity of in vitro differentiated NCR^+^ILC3s in cell culture was verified by sorting (**A**) and subsequent functional analysis, including flow cytometry (**B**). The overlapping histograms display stained cells (empty curve) over unstained cells (gray filled area). (**C**) NCR^+^ILC3 cell numbers remain unchanged after treatment with 100 IU/mL hCG, 2 ng/mL TGF-β1 and different oxygen concentrations. Bars show mean ± SEM normalized to the media of untreated cells. Data was analyzed by paired Student’s *t*-test. The experiment was repeated 5 (8 and 1% O_2_) or 10 times (21% O_2_) in duplicates.

## Data Availability

Not applicable.

## Abbreviations

ILCs: Innate lymphoid cells
MHC: Major histocompatibility complex
NCR: Natural cytotoxicity receptor
hCG: Human chorionic gonadotropin
TGF-β1: Transforming growth factor β1
HLA: Human leukocyte antigen
NK: Natural killer
BAFF: B-cell activating factor
DLL1: Delta like canonical notch ligand 1
CD: Cluster of differentiation
IL: Interleukin
SCF: Stem cell factor
Flt-1: Fms-Like Tyrosine Kinase 1
HIF-1ɑ: Hypoxia-inducible factor 1-alpha
FACS: Fluorescence-activated cell sorting
BSA: Bovine serum albumin
DPBS: Dulbecco’s phosphate-buffered saline
FMO: Fluorescence minus one
RORγt: RAR-related orphan receptor gamma
SEM: Standard error of mean
MFI: Mean fluorescence intensity
