# Supplementary figures and images for: Oxygen regulates ILC3 antigen presentation potential and pregnancy-related hormone actions
Source: Reprod Biol Endocrinol. 2022 Jul 29;20:109. doi: 10.1186/s12958-022-00979-2 (PMC9336067; doi:10.1186/s12958-022-00979-2)

a

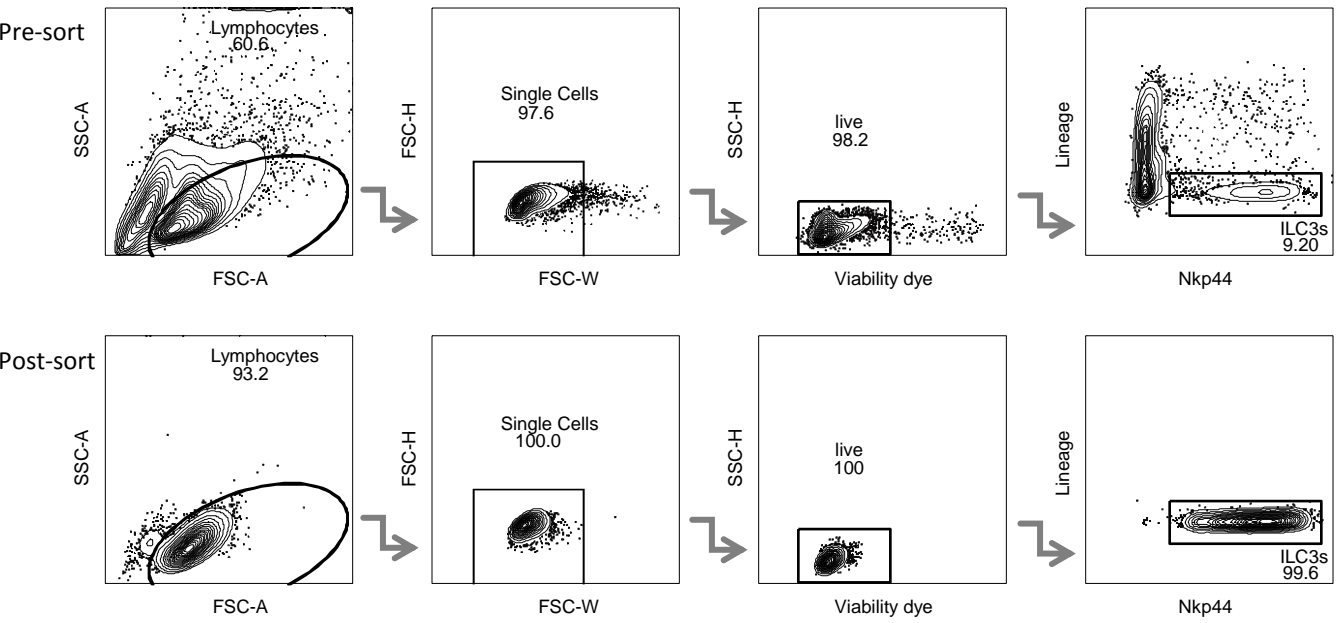

b

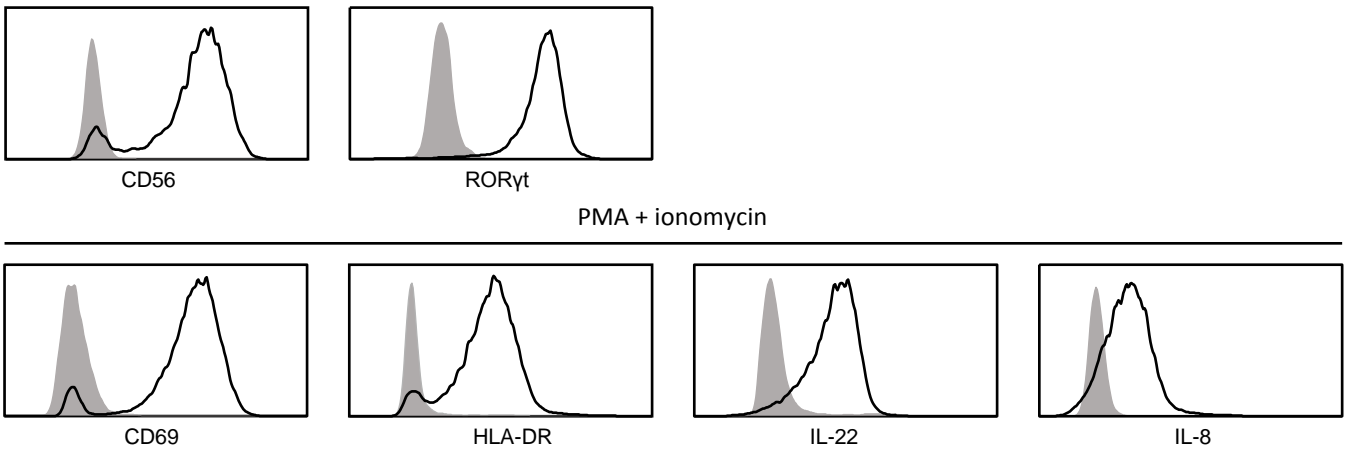

c

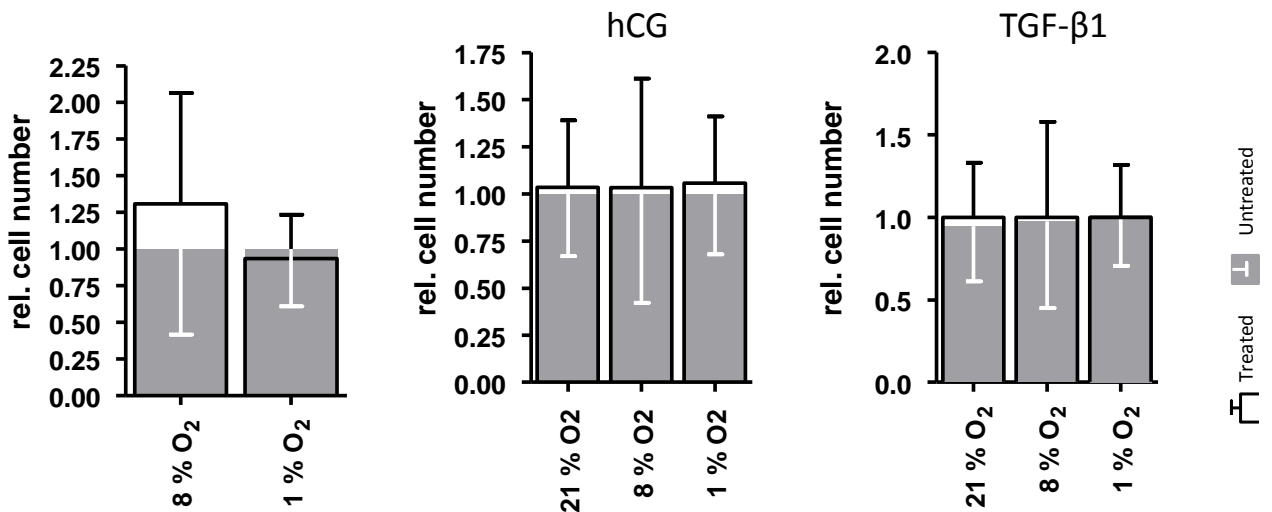

Supplement: Supplementary file 1 — Additional file 1: Supplementary figure. The identity of in vitro differentiated NCR+ILC3s in cell culture was verified by sorting (A) and subsequent functional analysis, including flow cytometry (B). The overlapping histograms display stained cells (empty curve) over unstained cells (gray filled area). (C) NCR+ILC3 cell numbers remain unchanged after treatment with 100 IU/mL hCG, 2 ng/mL TGF-β1 and different oxygen concentrations. Bars show mean ± SEM normalized to the media of untreated cells. Data was analyzed by paired Student’s t-test. The experiment was repeated 5 (8 and 1% O2) or 10 times (21% O2) in duplicates. [file 12958_2022_979_MOESM1_ESM.pdf]
